# A five-year retrospective study of patient falls in a tertiary hospital: frequency, characteristics, and contributing factors

**DOI:** 10.1186/s12913-026-14084-2

**Published:** 2026-01-28

**Authors:** Nazife Öztürk, Hatice Esen Koç

**Affiliations:** Research and Development Department, Antalya Training and Research Hospital, Antalya, Türkiye

**Keywords:** Falls in hospitals, Patient falls, Patient safety, Incident reporting

## Abstract

**Background:**

Falls in hospitalized patients are among the most common preventable adverse events and pose a major threat to patient safety. Despite preventive protocols, falls remain frequent and are associated with increased morbidity, prolonged hospitalization, and higher healthcare costs. Understanding the frequency, characteristics, and contributing factors of falls is essential to improve patient safety strategies.

**Aim:**

This study aimed to examine the frequency and characteristics of inpatient falls over a five-year period and to identify conributing factors and reported causes based on adverse event notifications.

**Methods:**

A retrospective descriptive study was conducted using hospital adverse event notification records between 2020 and 2024. A total of 211 fall incidents were identified and analyzed in terms of demographic characteristics, clinical settings, fall locations, risk scores, and root causes. The Itaki Fall Risk Scale was used to assess patient risk levels, and contributing factors were categorized into patient-related, caregiver-related, equipment-related, environmental, staff-related, and procedural causes.

**Results:**

Among the 211 reported falls, the mean patient age was 50.4 ± 29.3 years, and most cases involved male patients (62.6%). The highest fall incidence was observed in 2024 (*n* = 78), indicating an increasing trend across the years. The majority of patients (92.4%) were classified as high risk according to the Itaki Fall Risk Scale. Most falls occurred in patient rooms (71.1%), followed by procedure/examination rooms (14.7%) and bathrooms/toilets (11.8%). Analysis of perceived causes reported by staff revealed that falls were primarily patient-related (59.7%), followed by caregiver-related (33.2%), equipment-related (4.7%), environmental factors (1.4%), and rarely staff- or procedure-related factors (0.5% each).

**Conclusion:**

Patient falls remain a significant safety concern in hospitals, with most cases occurring among high-risk patients and being preventable. The analysis of reported causes highlights the multifactorial nature of falls, emphasizing the need for comprehensive preventive strategies that address patient behavior, caregiver awareness, environmental safety, and staff compliance with protocols. Strengthening fall prevention programs is crucial to improving patient safety and reducing healthcare costs.

**Clinical trial number:**

Not applicable.

## Highlights

This study presents a five-year period and to identify conributing factors and reported causes based on adverse event notifications of inpatient falls in a tertiary training and research hospital.

The majority of falls occurred among high-risk patients aged 65–80 years and in male patients, with most events reported in patient rooms.

Patient-related factors, including cognitive impairment, polypharmacy, and lack of compliance with preventive measures, were identified as the primary causes of falls.

Despite preventive protocols, underreporting and insufficient patient/caregiver engagement remain critical challenges in fall prevention.

Findings emphasize the importance of systematic risk assessment, patient and caregiver education, and evidence-based fall prevention strategies to strengthen patient safety culture.

## Introduction

Hospitals serve as the cornerstone of healthcare institutions, playing a central role in the health ecosystem by providing uninterrupted healthcare services [1]. Ensuring patient safety is the foremost priority for achieving high-quality, effective, and safe patient care [2]. The World Health Organization (WHO) defines patient safety as the absence of preventable harm to a patient during the process of healthcare [3]. Patient safety has become the most important priority for hospitals, with efforts directed toward establishing safer care environments where major complications are reduced [4]. Consequently, quality improvement practices and patient safety initiatives have emerged as fundamental goals of hospital management, making the implementation of specific preventive measures essential to achieving these objectives [1].

Hospital falls are among the most common and preventable patient safety incidents worldwide. A fall is defined as an unintentional descent to a lower level, regardless of whether an injury occurs [5–7]. Falls represent a major global safety concern. The WHO reports that approximately 37.3 million falls each year require medical attention, and around 684,000 people die annually due to falls [8–10]. Notably, the incidence of falls increases with age, with one in three adults aged 65 years and older experiencing at least one fall annually [11, 12]. Furthermore, approximately 600,000 fall-related deaths occur worldwide each year, making falls one of the leading causes of traumatic mortality [13]. Falls are also recognized as a leading cause of preventable injuries [14]. To reduce this risk, the WHO recommends creating safer environments, enhancing community education, increasing fall-related research, and developing appropriate prevention policies [10].

The causes of hospital falls are multifactorial, involving patient characteristics, caregivers, environmental factors, medications, and deficiencies in care processes, all of which can contribute to an increased risk [6, 15]. Therefore, the use of risk assessment tools, the development of preventive protocols, and the understanding of contributing factors are essential strategies to improve patient safety in clinical settings [16–20].

Nurses play a central role in fall-prevention strategies, and effective nursing practices have been shown to directly reduce the incidence of falls [14, 17, 21–24]. Most research to date has focused on identifying fall risks within the framework of global imperatives for prevention and management; however, interventions and preventive strategies for falls have not yet provided a universally accepted gold standard [25]. In this context, identifying the frequency, causes, and risk factors of hospital falls is essential to guide the development of effective fall-prevention programs. This study aimed to examine the frequency and characteristics of inpatient falls over a five-year period and to reported circumstances and contributing factors.

## Methods

### Study design

This research was conducted as a descriptive study using retrospective data.

### Setting and sample

The study was carried out in a tertiary-level training and research hospital located in Antalya, Türkiye.

### Fall risk assessment tools

Identifying potential risk factors that may lead to patient falls during hospitalization is of critical importance [11]. According to the Turkish Ministry of Health’s Healthcare Quality Standards (HQS), risk assessments must be performed, fall incidents should be monitored, preventive measures should be implemented at patient, ward, and hospital levels, and outcomes should be followed using quality indicators [26, 27]. Within this framework, the Itaki Fall Risk Scale is used for adult patients, while the Harizmi Fall Risk Scale is applied to pediatric patients. The Itaki Fall Risk Scale evaluates 19 risk factors categorized as major or minor, and classifies patients into two groups: low risk and high risk [26, 28]. In this study, the risk levels derived from the Itaki Scale (low vs. high) were used for analysis.

### Data collection

Data were retrospectively obtained from patient records covering the years 2020–2024. Information was collected using the “Patient Fall Data Collection Form” and the “Patient Fall Data Analysis Form,” which are implemented under the HQS framework of the Ministry of Health. Over the five-year period, data from 211 inpatients who experienced a fall during hospitalization were included in the study.

- **Patient Fall Data Collection Form**: includes patient name, age, gender, diagnosis, clinical unit, fall location, fall risk score, cause of fall, and pre-/post-fall patient status.
- **Patient Fall Data Analysis Form**: a mandatory hospital reporting tool completed monthly, which documents the number of hospitalized patients, number of falls, fall rates, locations of falls, and distribution of risk scores. The fall rate was calculated using the following formula defined by the Ministry of Health [29]:

$${\mathrm{Fall}}\:{\text{Rate = }}\frac{{{\mathrm{Number}}\:{\mathrm{of}}\:{\mathrm{patient}}\:{\mathrm{falls}}}}{\begin{gathered}{\mathrm{Total}}\:{\mathrm{number}}\:{\mathrm{of}}\:{\mathrm{inpatients}} \hfill \\{\text{ + carried - over}}\:{\mathrm{patients}} \hfill \\{\text{ + outpatient}}\:{\mathrm{visits}} \hfill \\ \end{gathered} } \times {\mathrm{100}}$$

Data were analyzed using Statistical Package for the Social Sciences (SPSS) Statistics version 22. Descriptive statistics included means, standard deviations, and frequency distributions. Group differences were examined using Chi-square tests, with statistical significance set at *p* < 0.05.

### Ethical considerations

This study was approved by Antalya Traning and Research Hospital Ethics Committee (Approval No: 2025/188, dated 19.06.2025) and conducted in accordance with the Declaration of Helsinki. This study used retrospective, anonymized patient safety data. No direct patient contact was involved. The requirement for individual informed consent was waived by the Ethics Committee.

### Findings

**Fig. 1:**
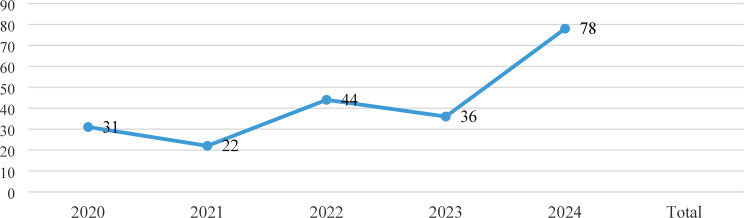
Distribution of the number of falls by year

During the five-year study period, a total of 211 inpatient falls were reported as adverse events. The lowest number of falls was observed in 2021 (*n* = 22), while the highest was recorded in 2024 (*n* = 78), indicating an upward trend over the years (Fig. 1).

During the five-year study period, a total of 211 inpatient falls were reported as adverse events. The lowest number of falls was observed in 2021 (*n* = 22), while the highest was recorded in 2024 (*n* = 78), indicating an upward trend over the years (Fig. 1).

The mean age of patients who experienced a fall was 50.43 ± 29.27 years, with the majority being male (62.6%). Falls were most frequently observed among patients in the 36–80 years age group (60.6%). The highest proportion of falls occurred in internal medicine clinics (43.1%), and according to the Itaki Fall Risk Scale, 92.4% of patients were classified as being at high risk for falls.

In terms of fall locations, the majority occurred in patient rooms (71.1%), followed by procedure/examination rooms (14.7%) and bathrooms/toilets (11.8%). The overall inpatient fall rate across the years was approximately 0.01%.

Evaluation of causes perceived and recorded by staff indicated that most falls (59.7%) were due to patient-related factors. Following the incidents, the majority of patients did not require additional diagnostic investigations (Table 1).

**Table 1:** Characteristics of patients who experienced falls (*n* = 211)

Variables	Mean ± SD/ *n* (%)		Min-Max
Age	50,43 ± 29,269		0–98
		n (211)	%
Gender	Female	79	37,4
	Male	132	62,6
Age	< 5	46	21,8
	6–17	3	1,4
	18–35	10	4,7
	36–64	64	30,3
	65–80	64	30,3
	> 81	24	11,4
Klinik	Emergency	54	25,6
	Surgical Clinics	24	11,4
	Internal Medicine Clinics	91	43,1
	Other	2	0,9
	Pediatric Clinics	40	19
Location of Fall	Clinic/Patient Room	150	71,1
	Examination/ Intervention Room	31	14,7
	Corridor	5	2,4
	Bathroom/toilet	25	11,8
Fall Risk Score	Low	16	7,6
	High	195	92,4
Causes of Falls	Patient - Related	126	59,7
	Caregiver - related	70	33,2
	Equipment - related	10	4,7
	Environment - related	3	1,4
	Staff - related	1	0,5
	Document - related	1	0,5
Additional Diagnostic Tests After Fall	No	196	92,9
	Yes	15	7,1
Fall Rate (per 1000 patients)	2020	31	0,014
	2021	22	0,008
	2022	44	0,015
	2023	36	0,011
	2024	78	0,023

**Fig. 2:**
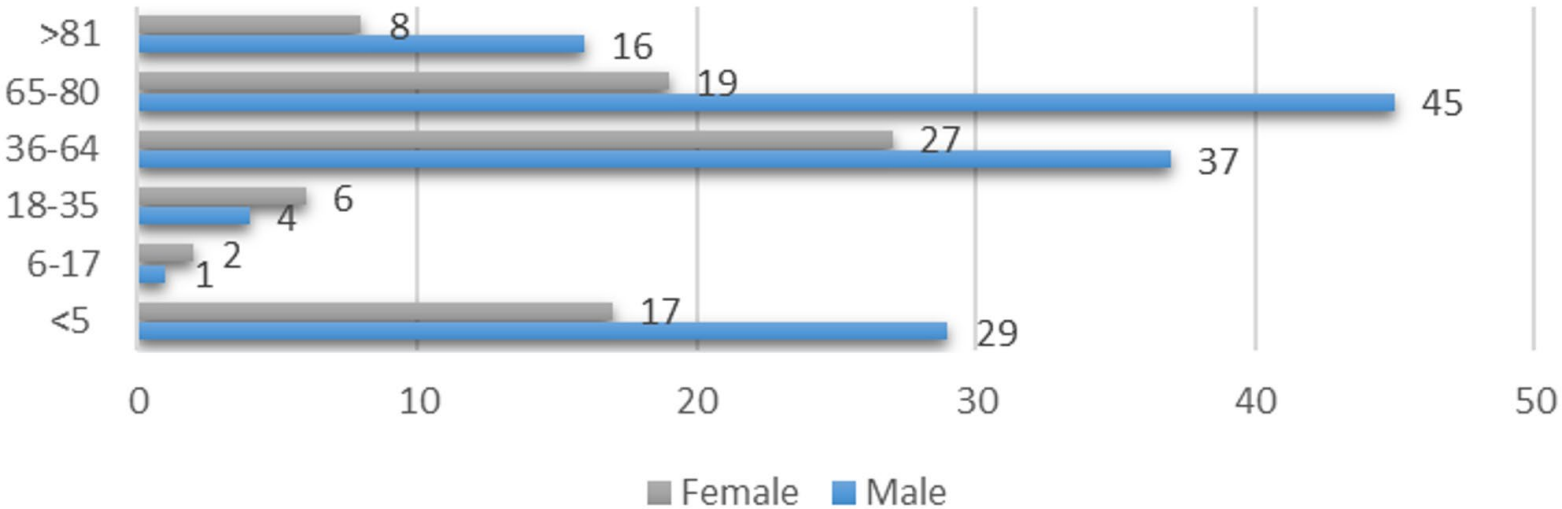
Gender distribution by age group

During the five-year study period, the distribution of patient falls by age and gender revealed that the highest number of falls occurred among male patients aged 65–80 years, followed by those in the 36–64 age group, and thirdly among children under the age of five (Fig. 2).

**Fig. 3:**
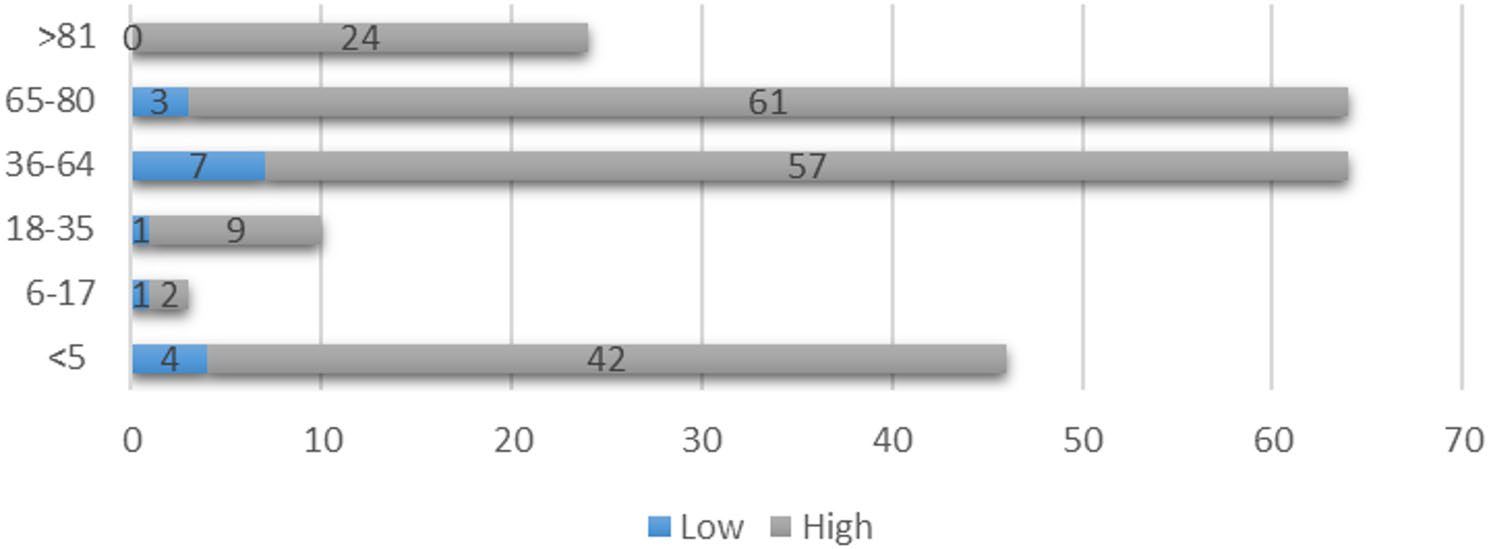
Distribution of fall risk (Low/High) by age group

When the distribution of fall risk scores (low–high) was examined by age group, the majority of patients were identified as being in the high-risk category, predominantly within the 65–80 age group (Fig. 3).

The association between gender and fall risk was analyzed using the Pearson chi-square test, and no statistically significant relationship was found (*p* = 0.194).

**Table 2:** Analysis of the relationship between age and fall risk

Age	Low	High	*p*
< 5	4	42	0.239
6–17	1	2	
18–35	1	9	
36–64	7	57	
65–80	3	61	
> 81	0	24	

The analysis of fall risk scores by age group demonstrated that the majority of patients across all age categories were classified as high risk. Specifically, in the 65–80 age group, 61 patients were identified as high risk compared to only 3 as low risk, while in patients older than 81 years, all cases (*n* = 24) were classified as high risk. Similarly, in the 36–64 age group, 57 patients were at high risk and only 7 at low risk. Among children under 5 years of age, 42 were categorized as high risk compared to 4 at low risk, whereas in the 6–17 age group, 2 patients were at high risk and 1 at low risk. The 18–35 age group also showed a predominance of high-risk cases (*n* = 9) compared to low-risk (*n* = 1). Statistical analysis using the Pearson Chi-Square test revealed no significant association between age and fall risk (*p* = 0.239). Despite the lack of statistical significance, the distribution indicates that older patients, particularly those aged 65 years and above, were more frequently classified as high risk, consistent with the literature highlighting advanced age as a major factor contributing to in-hospital falls (Table 2).

An analysis of reported contributing factors was performed for patient falls during the study period. Based on the analysis, the causes of falls were classified into six main categories: patient-related, caregiver-related, equipment-related, environment-related, staff-related, and procedure-related factors. The findings are summarized as follows:

Patient-related factors included agitation or anxiety, impaired consciousness, dizziness, loss of balance due to absence of a caregiver, attempts to sit or lie down independently, efforts to avoid disturbing their caregiver, parental attempts to sleep next to or rock their infant/child, wearing inappropriate slippers, orthostatic hypotension, hypoglycemia, polypharmacy, imbalance due to inability to use one leg, noncompliance with instructions, leaving the bed without permission, lowering bedrails independently, muscle weakness due to hemiplegia, and slipping of mobility aids.

Caregiver-related factors involved failure to comply with fall prevention measures, leaving the patient unattended without notifying nursing staff, absence of a caregiver, inadequate implementation of safety measures, allowing the patient to mobilize alone, distraction or negligence, falling asleep while holding a baby, not locking wheelchair brakes, and independently lowering bedrails or stretcher rails.

Equipment-related factors included unsuitable stretchers, rapid wear and tear of bed or stretcher side rails, and use of inappropriate slippers. Environment-related factors included inadequate lighting (e.g., room lights turned off), absence of wet floor warnings, and improper use of handrails. Staff-related factors involved being occupied with emergencies or caring for other patients, as well as errors in fall risk assessment. Procedure-related factors included procedural errors and improper implementation of existing protocols.

**Fig. 4:**
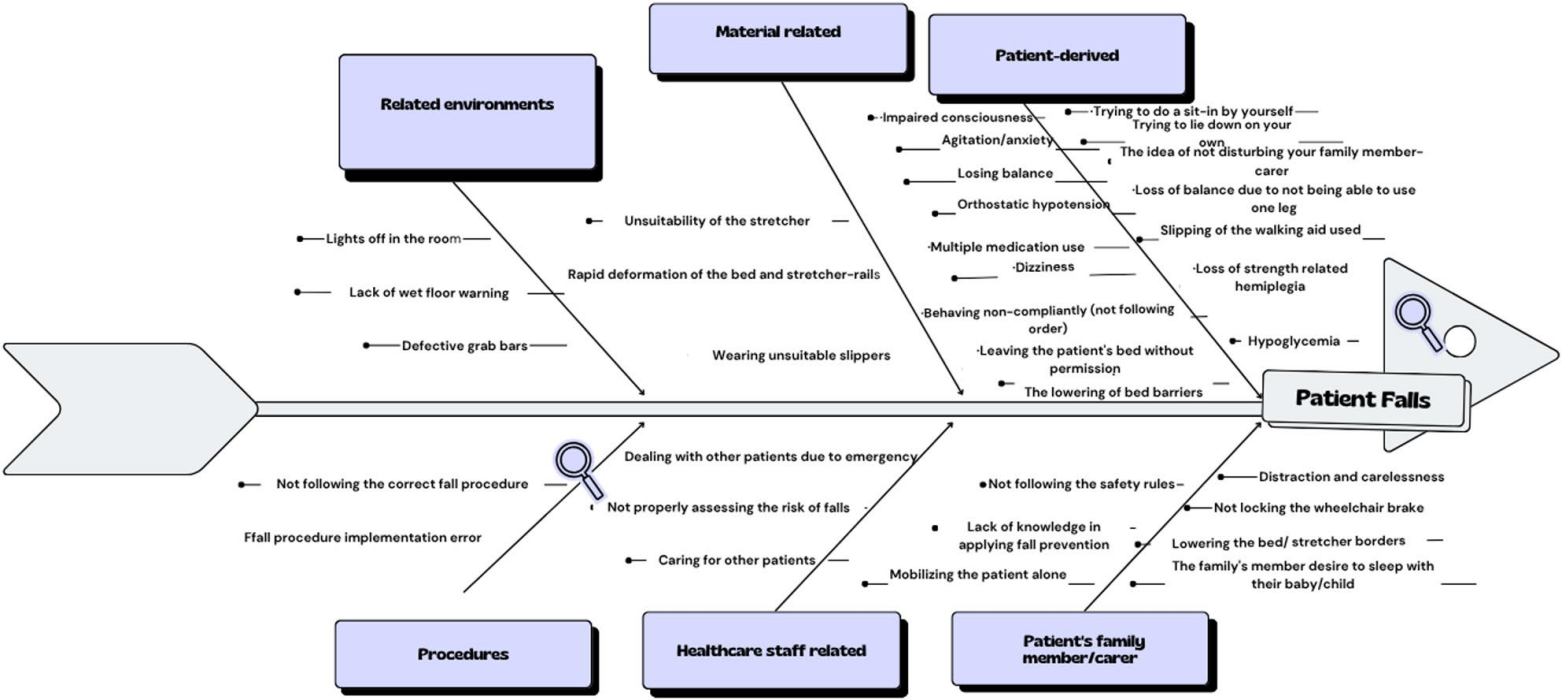
Fishbone diagram of contributing factors for patient falls

The analysis is presented in Fig. 4.

## Discussion

Patient safety aims to create a secure environment in which potential adverse events such as falls, medication errors, and infections are systematically evaluated [30]. In this context, the prevention of inpatient falls is of vital importance for ensuring the integrity of diagnostic and therapeutic processes [31]. Falls occurring in hospital settings are considered a critical indicator of patient safety [32, 30]. In this study, an analysis of contributing factors derived from incident reports of inpatient falls reported over a five-year period in a tertiary training and research hospital was conducted and evaluated. Although fall prevention interventions are effective, they require resources and should specifically target individuals at higher risk [20]. Efforts to reduce inpatient falls and their associated costs should prioritize patient engagement, education, and evidence-based interventions [33]. Hospital falls significantly prolong the length of stay (LOS), with reports indicating an additional 6 to 12 days of hospitalization following a fall [33, 34]. Similarly, falls have been associated with an average extension of 6.3 days in LOS [31].

Cognitive status plays a crucial role in increasing the likelihood of falls. A meta-analysis investigating hospital falls reported that 36.3% of patients who experienced falls presented with cognitive impairment [33]. In our study, while a precise prevalence rate for cognitive impairment could not be calculated due to the retrospective nature of incident reports (a limitation of this study), factors related to altered mental status—such as ‘impaired consciousness’, ‘agitation’, and ‘anxiety’—were frequently identified within the patient-related causes. It is aligns with the literature suggesting that cognitive and neurological instability are major contributors to falls, even if reporting mechanisms do not always capture formal psychiatric diagnosesProminent risk factors for inpatient falls include advanced age (> 85 years), male sex, fall history, gait instability, visual impairment, agitation and/or confusion, adverse drug reactions, neurological and cardiovascular instability, orthostatic hypotension, and pharmacological agents such as antihypertensives and diuretics [31, 33, 18, 35]. Previous studies have indicated that approximately 12% of hospitalized patients experience at least one fall during their stay [5].

Reported incidence rates vary, ranging from 2.4 per 1000 patient-days in tertiary hospitals to 9.1 per 1000 patient-days in geriatric units. In this study, the calculated fall rate was approximately 0.01 per 1,000 patients, which is exceptionally low compared to international benchmarks that typically range from 2.4 to 9.1 per 1,000 patient-days. This significant discrepancy strongly suggests the presence of underreporting. As highlighted by Cho et al. [23] incident reporting systems are often perceived by healthcare staff as ‘unsafe’ or punitive, leading to reluctance in reporting adverse events. It is likely that our data captures primarily those falls resulting in injury or requiring immediate intervention, while minor incidents or near-misses remain unreported. This finding emphasizes an urgent need for hospital management to foster a non-punitive, just culture to encourage transparent reporting.

Older adults with cognitive impairment are particularly vulnerable to falls [31]. Elderly people with cognitive impairment are more likely to slip and fall, which is a significant risk factor for falls [36]. According to Xu et al. (2022), factors such as advanced age, low educational level, polypharmacy, malnutrition, living alone, urban residency, smoking, and alcohol consumption increase the likelihood of falls in older adults. Additionally, comorbidities including cardiovascular disease, hypertension, diabetes, stroke, weakness, depression, Parkinson’s disease, and chronic pain are known contributors to fall risk [15, 33]. Studies have also examined the activities during which falls occur, such as attempting to sit, stand, bend, get out of bed, use the bathroom without assistance, or walk unassisted. Frequently reported locations of falls include bathrooms, patient rooms, treatment areas, hallways, and common hospital spaces [37].

In this study, fall risk was assessed using the Itaki Fall Risk Scale as mandated by the national Healthcare Quality Standards. However, it is important to contextualise this approach within recent global developments. The ‘World Guidelines for Falls Prevention’ [38] emphasise that relying solely on risk prediction scores (stratifying patients as simply low or high risk) may not be sufficient for effective prevention. Instead, these guidelines advocate for a shift towards a multi-disciplinary responsibility involving tailored interventions that address specific risk factors (e.g., medication review, environmental modification) rather than generic protocols based on a score. While the Itaki scale provides a necessary baseline for compliance, our findings support the need for such comprehensive, individualised strategies to further reduce fall rates.

The first step in fall prevention is the identification of high-risk patients and the implementation of targeted risk-reduction programs [31].

A non-punitive, supportive reporting culture is critical to ensure accurate reporting. Hospital administrators should adopt a visionary, non-punitive approach to promote confidence in reporting systems [1]. Underreporting remains a major issue, with estimates suggesting that approximately 40% of inpatient falls are not reported. In one study, nearly 75% of nurses in a tertiary hospital considered incident reporting systems unsafe [22].

The causes of hospital falls are often multifactorial, including patient-related factors (37.5%), environmental conditions (25%), organizational and process-related issues (19.6%), and staff communication problems (17.9%) [30]. In terms of external causes, Lakbala et al. identified environmental conditions (25%) and organisational issues (19.6%) as major contributors to hospital falls [31]. In contrast, our study found these factors to be minimal, with environment-related (1.4%) and staff-related (0.5%) causes constituting a very small fraction of reports. This discrepancy likely reflects the staff’s perception that falls are driven by patient behavior, potentially overlooking subtler environmental or systemic contributors (such as the management of delirium) that are less obvious at the moment of reporting. This underscores the need to focus prevention efforts not only on patient supervision but also on training staff to recognize underlying systemic triggers. This underscores the need to focus prevention efforts on patient supervision and education rather than solely on environmental modifications. Similarly, Cesar et al. (2025) reported that 76% of falls resulted from internal factors, while 21% were attributable to external causes [32]. Strategies for fall prevention include environmental modifications and physical safeguards (29.4%), risk assessment and monitoring (23.5%), patient and staff education (21.6%), standardized fall risk assessment tools (13.7%), and auditing and monitoring practices (11.8%) [30]. Evidence-based measures such as ensuring locked bed brakes, lowering bed height, providing bedside commodes, assisting patients during toileting, and hourly rounding have been identified as effective fall prevention strategies. However, qualitative studies have emphasized barriers including inconsistencies in guidelines, lack of patient awareness regarding fall risks, and inadequate interprofessional communication [33].

The consequences of inpatient falls include both physical and clinical outcomes. Approximately 59.2% of falls result in injuries, most commonly head injuries (49%). While some patients remain clinically stable post-fall, up to 20.4% experience instability [39]. Additional investigations, such as radiological imaging, are often required [32].

Falls are frequently linked to inadequate patient/family education, insufficient supervision, lack of communication between nurses and patients, ineffective use of call bells, and poor adherence to fall prevention guidelines. Multicomponent interventions that incorporate patient and caregiver training, environmental modifications, and nursing supervision have been shown to reduce fall rates [14].

Nurses play a pivotal role in the early identification and prevention of falls due to their continuous patient interaction [40]. As the frontline of patient care, nurses are essential in empowering patients and families with knowledge about fall prevention strategies such as using call bells, wearing safe footwear, utilizing assistive devices, and seeking help during mobilization [41]. While falls cannot be completely eliminated, effective nursing interventions can substantially minimize fall risks and adverse outcomes [40].

### Limitations and strengths

The present study acknowledged several strengths and limitations. A key strength of the study is its five-year longitudinal design, which allows for the monitoring of trends in fall characteristics and risk factors over a significant period. Additionally, the use of standardized data collection forms within the national Healthcare Quality Standards framework ensures data consistency and facilitates the identification of reported contributing factors.

Nevertheless, this study is not without its limitations. First and most critically, the fall rate calculated in this study is notably lower than international benchmarks. This discrepancy strongly suggests a likelihood of underreporting, where staff may be reluctant to report incidents due to a fear of potential repercussions or workload constraints. Consequently, the findings should be interpreted as the incidence of reported falls rather than the absolute prevalence of all fall incidents. Second, as study data were extracted solely from adverse event notifications rather than comprehensive clinical records, specific clinical variables—such as the precise prevalence of cognitive impairment among patients who fell—could not be quantitatively assessed, limiting direct comparisons with some external studies. Finally, the reliance on retrospective incident reports rather than in-depth qualitative investigations restricts the analysis to reported contributing factors, rather than establishing deep root causes.

Notwithstanding these limitations, the study’s findings provide significant insights into the characteristics of falls in high-risk groups and highlight the urgent need to improve the safety reporting culture within healthcare environments.

## Conclusion

This five-year retrospective study revealed an increasing trend in inpatient falls, predominantly affecting male patients aged 65–80 years, with the majority of incidents occurring in patient rooms. Although adherence to risk assessment protocols was high, with 92.4% of patients correctly identified as “high risk” before the fall, this categorisation alone was insufficient to prevent incidents. The analysis of contributing factors indicated that falls were primarily driven by patient-related behaviours (59.7%), such as unassisted mobilisation attempts, rather than equipment failure. Furthermore, the exceptionally low fall rate observed in this study compared to international benchmarks suggests a critical need to address underreporting and barriers within the safety reporting culture. Consequently, future prevention strategies should move beyond standard risk scoring to include tailored, multi-disciplinary interventions and administrative efforts to foster a transparent, non-punitive reporting environment.

## Data Availability

The data from the current study are available from the corresponding author upon reasonable request.

## Abbreviations

WHO: Worl Health Organizations
HQS: Healthcare Quality Standards
LOS: Length of Stay
SPSS: Statistical Package for the Social Sciences
SD: Standart Deviation
